# Comparison of Use Rates of Telehealth Services for Substance Use Disorder During and Following COVID-19 Safety Distancing Recommendations: Two Cross-Sectional Surveys

**DOI:** 10.2196/52363

**Published:** 2024-08-12

**Authors:** Adrijana Pusnik, Bryan Hartzler, Olivia Vjorn, Beth A Rutkowski, Michael Chaple, Sara Becker, Thomas Freese, Maureen Nichols, Todd Molfenter

**Affiliations:** 1 Center for Health Enhancement Systems Studies (CHESS), Department of Industrial and Systems Engineering, University of Wisconsin-Madison, Madison, WI, United States; 2 Addictions, Drug & Alcohol Institute, Psychiatry and Behavioral Sciences, University of Washington School of Medicine, Seattle, WA, United States; 3 Integrated Substance Use and Addiction Programs, Division of Addiction Psychiatry, University of California, Los Angeles, Los Angeles, CA, United States; 4 New York State Psychiatric Institute, Division of Substance Use Disorders, Columbia University Irving Medical Center, New York City, NY, United States; 5 Center for Dissemination and Implementation Science, Northwestern University Feinberg School of Medicine, Chicago, IL, United States; 6 Addiction Research Institute, Steve Hicks School of Social Work, University of Texas at Austin, Austin, TX, United States

**Keywords:** telehealth, COVID-19, substance use disorders, telephone counseling, video counseling

## Abstract

**Background:**

The COVID-19 social distancing guidelines resulted in a dramatic transition to telephone and video technologies to deliver substance use disorder (SUD) treatment. Before COVID-19, the question was “Will telehealth ever take hold for SUD services?” Now that social distancing guidelines have been lifted, the question is “Will telehealth remain a commonly used care modality?”

**Objective:**

The principal purpose of this investigation was to examine the extent to which telehealth use in SUD service settings persisted following the lifting of COVID-19 safety distancing recommendations. Additionally, the study aimed to explore practitioners’ perceptions of telehealth convenience and value after its regular implementation during the pandemic. Specifically, the goal of this study was to compare telehealth activity between time intervals: May-August 2020 (during peak COVID-19 safety distancing recommendations) and October-December 2022 (following discontinuation of distancing recommendations). Specifically, we compared (1) telehealth technologies and services, (2) perceived usefulness of telehealth, (3) ease of use of telephone- and video-based telehealth services, and (4) organizational readiness to use telehealth.

**Methods:**

An online cross-sectional survey consisting of 108 items was conducted to measure the use of telehealth technologies for delivering a specific set of SUD services in the United States and to explore the perceived readiness for use and satisfaction with telephonic and video services. The survey took approximately 25‐35 minutes to complete and used the same 3 sets of questions and 2 theory-driven scales as in a previous cross-sectional survey conducted in 2020. Six of 10 Regional Addiction Technology Transfer Centers funded by the Substance Abuse and Mental Health Services Administration distributed the survey in their respective regions, collectively spanning 37 states. Responses of administrators and clinicians (hereafter referred to as staff) from this 2022 survey were compared to those obtained in the 2020 survey. Responses in 2020 and 2022 were anonymous and comprised two separate samples; therefore, an accurate longitudinal model could not be analyzed.

**Results:**

A total of 375 staff responded to the 2022 survey (vs 457 in 2020). Baseline organizational characteristics of the 2022 sample were similar to those of the 2020 sample. Phone and video telehealth utilization rates remained greater than 50% in 2022 for screening and assessment, case management, peer recovery support services, and regular outpatient services. The perceived usefulness of phone-based telehealth was higher in 2022 than in 2020 (mean difference [MD] −0.23; *P*=.002), but not for video-based telehealth (MD −0.12; *P*=.13). Ease of use of video-based telehealth was perceived as higher in 2022 than in 2020 (MD−0.35; *P*<.001), but no difference was found for phone-based telehealth (MD −0.12; *P*=.11). From the staff’s perspective, patients had greater readiness for using telehealth via phone than video, but the staff perceived their personal and organizational readiness for using telehealth as greater for video-based than for phone-based telehealth.

**Conclusions:**

Despite lower telephone and video use in 2022 for telehealth services than in 2020, both modalities continue to be perceived positively. Future research may further determine the relative cost and clinical effectiveness of video-based services and thereby help to address some sources of the noted challenges to implementation by SUD organizations.

## Introduction

Use of telehealth, defined as the remote provision of health services via telecommunications, has proliferated over the past decade [1]; however, its uptake has been complex and inconsistent [2]. Despite compelling evidence that telehealth services result in equal or better clinical effectiveness and patient satisfaction relative to in-person services across multiple meta-analytic reviews [3-7], telehealth use had been extremely limited in the delivery of services for substance use disorders (SUDs). One national estimate in 2018 cited the use of telehealth in only 16% of SUD treatment programs [8]. Following the onset of COVID-19 in 2020, the implementation of corresponding safety distancing recommendations created what some have termed a disruptive innovation scenario [9] wherein the use of telephone- and video-based telehealth services was urgently supported at a federal level and began to occur among treatment providers at unprecedented levels [10].

The unforeseen circumstances of the global pandemic brought an opportunity for greater understanding of telehealth use among treatment organizations offering SUD services. Among the encouraging findings reported in the wake of new COVID-19 safety policies was the increased use of telehealth in engaging patients to receive medications for opioid use disorders, which was associated with greater treatment retention and a reduced chance of medically treated overdose [11]. Similarly, telehealth services in the midst of COVID-19 safety distancing recommendations left a favorable impression among clinical and administrative staff in terms of the clinical benefit and reach of vulnerable populations [12-14]. An additional related benefit was the perception of telehealth’s favorable influence in both reducing SUD stigma and increasing support for patients in active recovery [15,16].

Concerns with telehealth also emerged during COVID-19. Multiple reports noted a greater acceptability of and preference for in-person services among some staff and patient groups [5,17], while others detailed concerns regarding the lack of human contact, confidentiality, and data security when using telehealth [18]. Perhaps unsurprisingly, the eventual loosening of COVID-19 safety distancing recommendations was reportedly associated with a significant reduction in telehealth use [10]. Taken together, a muddied picture of equivocal and rapidly shifting findings casts some doubt on what rates of telehealth use may be expected as treatment organizations proceed through the post-COVID-19 era.

Based on a national sampling of administrators and clinicians from 457 SUD treatment organizations in the United States in the months immediately following the institution of COVID-19 safety distancing recommendations (May to August 2020), a group affiliated with the Substance Abuse and Mental Health Services Administration (SAMHSA) Addiction Technology Transfer Center (ATTC) Network reported several findings concerning telehealth utilization by the addiction treatment community [19]. Foremost among these were: (1) a pattern of extensive telehealth (via telephone and video) utilization by 73% of the SUD treatment organizations, with its most prevalent application in screening and assessment intake (79%) and general outpatient services (82%); (2) strong organizational readiness to use, and satisfaction with, both telephone- and video-based technologies, albeit with the former technology deemed more accessible among patients with SUDs; and (3) validation of the Technology Acceptance Model (TAM) [20] in SUD service settings such that, for both telephone- and video-based technologies, perceived usefulness and ease of use predicted the organizational intent for continued utilization following the discontinuation of COVID-19 safety protocols. The current work offers a follow-up investigation of this 2020 survey using a similar cross-sectional national sampling of personnel from SUD treatment organizations from October to December 2022. The primary aim of this study was to examine the extent to which telehealth use in SUD service settings persisted following discontinuation of COVID-19 safety distancing recommendations. Specifically, we compared use rates for specific telehealth technologies and services, the perceived usefulness of telehealth, ease of use of telephone- and video-based telehealth services, and organizational readiness to use telehealth between the two time intervals: May-August 2020 (peak COVID-19 distancing recommendations) and October-December 2022 [1] (following discontinuation of distancing recommendations).

## Methods

### Study Design

The voluntary cross-sectional online survey (described in further detail below in the Survey Instrument subsection) to measure the use of telehealth technologies for delivering a specific set of SUD services and to explore the perceived readiness to use and satisfaction with telephonic and video services was developed using similar questions as our prior telehealth survey distributed during the peak of social distancing recommendations [1]. Six of 10 regional ATTCs distributed the survey to administrators, clinicians, and recovery personnel in their respective regions. The distribution collectively spanned 37 states. SAMHSA-funded ATTCs support the workforce for addiction treatment and recovery via regional ATTCs that correspond to the 10 regional offices of the US Department of Health and Human Services. Four regional ATTCs chose not to participate, citing concerns about potential survey fatigue among their stakeholders.

### Data Collection

The 2022 survey was distributed on October 3, 2022, and data collection continued until January 6, 2023; the survey took approximately 8‐15 minutes to complete [21]. Regional ATTCs distributed survey links to administrators and clinicians at substance use treatment organizations (hereafter referred to as “staff” for simplicity) via their regional mailing lists.

### Survey Instrument

The survey instrument had 104 questions that included 3 sets of study-specific questions, designed to mimic the questions used in the 2020 survey, followed by 2 theory-driven scales. The method used to test survey usability and validity is described in Molfenter et al [19]. The first set of questions asked about the organization where the respondent worked. Specifically, respondents were asked to select their organization type (ie, health system, opioid treatment program, recovery community organization, and specialty addiction treatment providers such as nonopioid treatment programs), organization location (ie, tribal reservation, rural, small city, suburban, and urban), and organizational role (ie, administrators and personnel providing treatment and/or recovery services).

The second set of questions assessed the organization’s use of the following technologies via binary yes/no variables: computerized screening and assessments, mobile app(s) during recovery, mobile app(s) during treatment, web portal for scheduling appointments, secure chats for recovery support sessions, text appointment reminders, text motivational messages, and video-based therapy to provide buprenorphine.

The third set of questions asked respondents which methods (telephone, video, or in person) were used for the following services: screening and assessment, buprenorphine therapy, case management, intensive outpatient treatment, peer recovery support, regular outpatient treatment, and residential counseling sessions.

The 2 theory-driven scales followed the above questions based on the Technology Acceptance Scale [19], which includes two subscales from the TAM: ease of use and perceived usefulness [20]. The ease-of-use scale assesses the ease of learning, customizing, and using a technology. Perceived usefulness assesses the extent to which the technology is perceived to enhance effectiveness, improve performance, increase productivity, and be useful. Items in these subscales were scored on 5-point Likert scales with endpoints of 1=“strongly disagree” and 5=“strongly agree.”

The Organizational Readiness for Technology Use predictive tool was used to assess dimensions of organizational readiness for the use of telephone and video technologies [22,23]. Each item was evaluated using a 5-point Likert scale with endpoints of 1=“strongly disagree” and 5=“strongly agree.” The inventory assessed the perceived feasibility of reimbursement for the technology during and after COVID-19; access to information technology experts, clinical champions, and billing experts to support the use of these technologies; ease of technology integration into the workflow; staff, facilities, and equipment to promote the technology; leadership, staff, and patient support; technology accessibility and affordability; and staff training.

### Data Analysis

Responses from the 2020 cross-sectional survey were compared to responses in the 2022 cross-sectional survey. Since the responses in 2020 and 2022 were anonymous and comprised two separate samples, a true longitudinal model could not be analyzed. Each staff member answered the same set of telephone and video telehealth questions. We calculated frequencies and descriptive statistics for the questions about participating site characteristics and use of different telehealth services. To compare the different services used between 2020 and 2022, we analyzed each service separately and removed organizations that did not offer a particular service from that specific analysis. The following model comparison between the years 2020 and 2022 was performed using the glmer() function with a binomial distribution since “yes/no” binomial outcomes were present in the data:

outcome = β_0_ + β_1_year + ν_0_ + e,

with ν_0_ representing the organization’s random effects.

For models where each staff member had multiple responses, the telehealth type (telephone vs video) varied among and within staff members and organizations. Thus, responses were nested in staff members and staff members were nested within organizations. Using the methods described by Brauer and Curtin [24], the following random effects structure was used:

outcome = β_0_ + β_1_type + β_2_year + β_3_type × year + u_0_ + u_1_type + ν_0_ + ν_1_type + e,

where u is the random intercept for organizations and e is the within-organization random error.

### Ethical Considerations

The University of Wisconsin’s Health Science Minimal Risk Institutional Review Board conducted a review of the study (2020‐0551) and determined that it met the criteria for exempt human subjects research in accordance with the definition under 45 Code of Federal Regulations (CFR) 46 [25]. No incentive was provided to the respondents to complete the questionnaire and responses were collected using Research Electronic Data Capture (REDCap), a secure web application. The University of Wisconsin’s institutional review board approved the presurvey information sheet, survey distribution, and recruitment of study participants.

## Results

### Basic Characteristics of the Samples

A total of 511 participants started the survey and 136 were removed in total (76 for incomplete responses, 2 that were part of our staff testing, and 8 for duplicate responses by the same individual). An incomplete response was removed if less than 4 of the 12 survey scales or sections (with demographics counting as one scale) were completed. For those who completed 4 or more of the sections, only completed sections were used in the relevant analyses. Duplicate responses were determined by matching email identifiers: only the first complete response was retained in cases of duplicate emails. IP addresses were not used due to shared staff computers.

The final sample in the 2022 survey included 375 responses from 325 unique organizations located across 37 states. The survey was distributed to 2102 organizations that provide SUD services, with an estimated return rate of 15% (325 organizations). The staff members predominantly reported their setting as an urban environment (177/375, 47.2%; see Table 1). The distribution of responses between organizational settings was not significantly different from that of the 2020 sample (*χ*^2^_3_=4.48, *P*=.21). There was a fairly even distribution of staff members across the organizational types in 2022, with the most respondents (58%) being from a specialty treatment setting (23% for stand-alone sites and 2‐5 sites, and 12% for 5+ organizational sites; see Table 1). The characteristics related to organizational type did differ significantly from those in 2020 (*χ*^2^_4_=13.34, *P*=.01) due to a higher percentage of participants from the recovery community in 2022. The distribution of staff members’ roles within the organization also significantly differed between 2020 and 2022 (*χ*^2^_1_=8.38, *P*=.004), with a higher percentage of staff members in 2022 reporting that they provided treatment and/or recovery services than in 2020. To account for the differences in the samples, organization type and staff members’ roles within the organization were added to the models comparing responses from 2020 and 2022.

**Table 1.** Characteristics of the participating organizations and staff.

Characteristic		Participating staff members in 2020 (n=581), n (%)	Participating staff members in 2022 (n=370), n (%)	*χ* ^2^	*df*	*P* value
**Organization setting**				4.48	3	.21
	Urban	244 (42)	177 (48)			
	Suburban	101 (17)	56 (15)			
	Small city	119 (21)	62 (17)			
	Rural	117 (20)	67 (18)			
	Tribal reservation	―^a^	8 (2)			
**Organization type**				13.34	4	.01
	Health system (Hospital, HMO^b^, or primary care network)	101 (19)	53 (14)	2.42	1	.12
	Specialty behavioral health: stand-alone	121 (22)	84 (23)	0.02,	1	.90
	Specialty behavior health provider: at 2-5 sites^c^	211 (39)	83 (23)	1.54	1	.21
	Specialty behavior health provider: 5+ sites^c^	―	44 (12)	1.54	1	.21
	Opioid treatment program	59 (11)	41 (11)	<0.01	1	.97
	Recovery community	52 (10)	63 (17)	10.71	1	.001
**Role within the organization**				8.38	1	.004
	Administrator	205 (37)	103 (28)			
	Personnel providing treatment and/or recovery services	344 (63)	266 (72)			

a Not a response option in the 2020 survey.

b HMO: Health Maintenance Organization.

c These two categories were combined to compare to 2020.

### Technology Use

The probability of using various technologies did not change from 2020 to 2022, except for the use of secure chats for recovery support sessions (*P*=.02; see Table 2). In 2020, SUD treatment staff were more likely to use secure chats for recovery support sessions than in 2022 (Table 2).

**Table 2.** Use of different technologies.

Technology	Probability (95% CI)		*P* value
	2020	2022	
Computerized screening and assessments	0.76 (0.68‐0.82)	0.80 (0.71‐0.87)	.24
Mobile app(s) during recovery	0.00 (0.00‐0.00)	0.00 (0.00‐0.00)	.89
Mobile app(s) during treatment	0.24 (0.17‐0.31)	0.18 (0.12‐0.27)	.14
Organizational web portal patients can use to schedule appointments	0.12 (0.07‐0.18)	0.16 (0.10‐0.24)	.10
Secure chats for recovery support sessions	0.44 (0.38‐0.51)	0.33 (0.26‐0.42)	.02
Text appointment reminders	0.60 (0.51‐0.68)	0.68 (0.58‐0.77)	.07
Text motivational messages	0.00 (0.00‐0.00)	0.00 (0.00‐0.00)	.93

### Use of Different Telehealth Services

**Figure 1.**
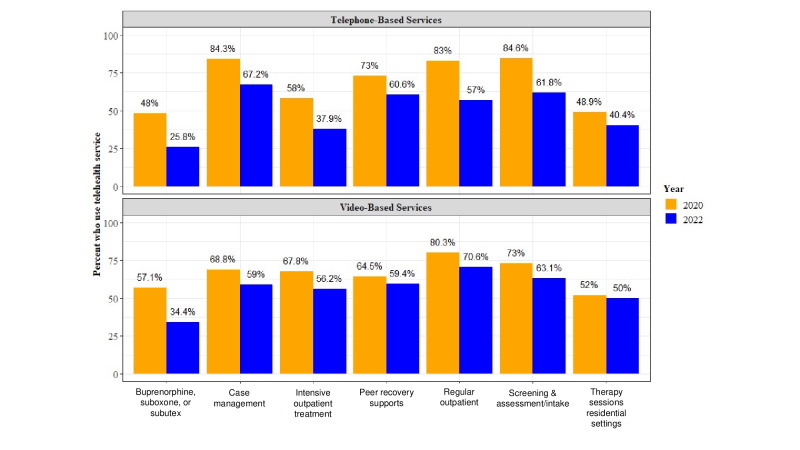
Telehealth services availability level (2020 and 2022).

Overall, the respondent organizations’ use of telephone- and video-based telehealth services significantly declined between 2020 and 2022, as shown in Figure 1 and Table 3. The only exceptions were video-based peer recovery services (*P*=.34) and video- and telephone-based therapy services in residential treatment programs (*P*=.79), all of which also declined but not significantly (Table 3). These data present a mixed picture (see Figure 1). Telehealth use remained encouragingly high (greater than 50%) for screening and assessment, case management, peer recovery support, and regular outpatient services, yet the utilization of these services significantly declined since the early months of the COVID-19 distancing recommendations (Figure 1 and Table 3).

**Table 3.** Use of different telehealth services for 2020 versus 2022.

Telehealth services	Telephone-based service		Video-based service	
	*χ*^2^ (*df*=1)	*P* value	*χ*^2^ (*df*=1)	*P* value
Buprenorphine, suboxone, or subutex	15.39	<.001	15.36	<.001
Case management	22.91	<.001	5.44	.02
Intensive outpatient treatment	14.53	<.001	5.09	.02
Peer recovery support	6.85	.009	0.92	.34
Regular outpatient	50.6	<.001	7.51	.006
Screening and assessment/intake	45.11	<.001	7.22	.007
Therapy session residential settings	2.12	.14	0.07	.79

### Perceived Usefulness and Ease of Use

**Figure 2.**
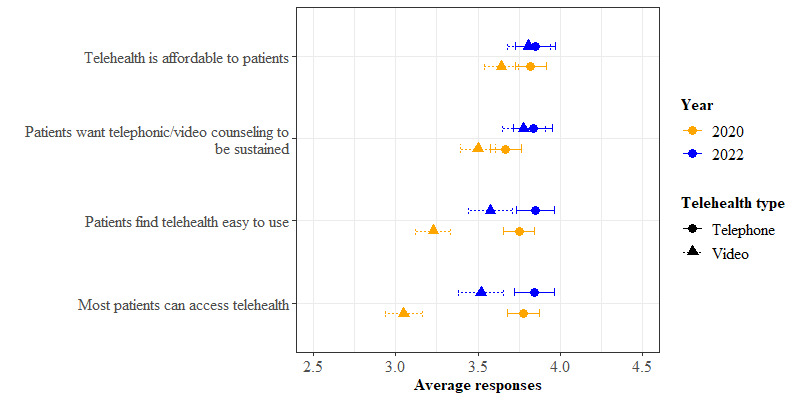
Staff responses regarding the patient’s perspective.

Overall, video was perceived as more useful for telehealth than the phone (mean difference [MD] 0.16; *P*<.001) across the 2020 and 2022 samples (Figure 2). Telehealth (phone and video together) was perceived as more useful in 2022 than in 2020 (MD 0.23; *P*=.002). This overall effect was driven by a change in perceptions of telephone-based telehealth, for which perceived usefulness was higher in 2022 than in 2020 (MD −0.23; *P*=.002); however, there was no significant difference between years in the perceived usefulness of video-based telehealth (MD −0.12; *P*=.13).

By contrast, telephone was perceived as easier to use for telehealth than video (MD=−0.32, *P*<.001) across both the 2020 and 2022 samples. There was no statistically significant difference between 2020 and 2022 in perceived ease of use for telehealth (telephone and video together) (MD 0.12; *P*=.11). For video-based telehealth, ease of use was perceived higher in 2022 than in 2020 (MD −0.35; *P*<.001), whereas for telephone-based telehealth, there was no significant difference in ease of use between 2022 and 2020 (MD −0.12; *P*=.11).

### Organizational Readiness for Using Telehealth

**Figure 3.**
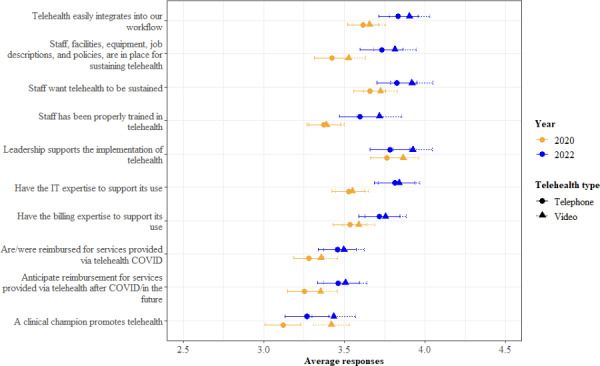
Staff responses regarding their personal and organizational readiness.

Staff responses regarding organizational readiness showed that from their perspective, patients had greater readiness for using telehealth via telephone than via video (Figure 2). By contrast, staff perceived their personal and organizational readiness for using telehealth as greater for video than for the telephone, as shown in Figure 3. Staff ratings of both patient and organizational readiness for telehealth were higher in 2022 than in 2020.

This overall effect was driven by a change in perceptions of telehealth via video. Staff reported that patients found video telehealth easier to use in 2022 than in 2020 (MD −0.35; *P*<.001). In contrast, there was no difference in perceptions of ease of use of telephone-based telehealth between years (MD −0.10; *P*=.19). Additionally, from the staff’s perspective, more patients had greater access to video telehealth in 2022 than in 2020 (MD −0.47; *P*<.001).

## Discussion

The goal of this study was to compare the availability, perceived usefulness, and ease of use of telehealth services across two time intervals: during peak COVID-19 distancing requirements in 2020 and following the removal of distancing requirements in 2022 [19]. Relative to 2020, there was a notable decrease in the use of telehealth services across various clinical tasks, targets, and programs (ie, screening and assessment, case management, regular and intensive outpatient programs, medications for opioid use disorder), with telephonic services experiencing a more pronounced decline in utilization compared to video-based services. Despite the decline in use of telehealth services from 2020 to 2022, the majority (57%‐71%) of the 325 SUD organizations surveyed in 2022 reported the continued utilization of telehealth services, a rate that was much higher than national estimates, suggesting that 20%‐30% of SUD organizations offered telehealth prior to the COVID-19 pandemic [6]. The persistent use of telehealth services in 2022 highlights the popularity of the regulatory flexibilities that were implemented during the COVID-19 pandemic: such flexibilities were initially intended to be temporary but have endured due to substantial advocacy efforts led by providers, patients, investors, and policy makers [26-33].

The current findings replicated the patterns revealed in the 2020 survey reported by Molfenter et al [1], highlighting the perceived usefulness of video-based services and the perceived ease of use of telephonic services. Notably, while both effects were consistent with the prior report, they were of lesser magnitude following the discontinuation of COVID-19 safety protocols. Prior research indicates strong patient satisfaction with video-based services [34], either greater than or equivalent to satisfaction with telephone services. However, confidence in these results is limited by the lack of well-validated measures assessing satisfaction with technology: a 2022 systematic review identified 10 scales across 12 studies, finding 9 of the studies to be of “inadequate” or “doubtful” quality. Furthermore, the scalability of video-based services presents significant challenges. Cost is a major issue, encompassing the initial purchase and ongoing operation of videoconferencing platforms and securing reimbursement from state Medicaid systems and insurers. Another challenge is protecting patient anonymity and complying with the Health Insurance Portability and Accountability Act and 42 CFR Part 2, which provides privacy protections for SUD-related records [35,36]. The absence of an accreditation system for documenting compliance in telehealth service delivery leaves individual organizations in a precarious position concerning the 42 CFR regulatory requirements [37].

Another reason that video might consistently be rated as more difficult to use is organizational resistance encountered when unfamiliar technologies are incorporated into familiar staff roles, functions, and workflow. Fortunately, this challenge may be addressed via workforce education efforts. To that end, the ATTC Network has, in recent years, developed learning resources to aid this cause. One example is demonstration videos, which, as asynchronous learning resources, enable workforce members to individually access and observe models to visualize and approximate their future clinical practice behavior. Among available ATTC-sponsored demonstration videos are those modeling the video-based delivery of (1) empirically supported therapies (eg, motivational interviewing, cognitive behavioral therapy), (2) care interactions in the context of medications for opioid use disorder, and (3) effective clinical supervision practices. Several user-friendly resources to promote the use of telehealth for delivering evidence-based SUD practices can be found on the ATTC Network website [38]. Beyond workforce education efforts, a salient priority within the addiction research community should be further validation of the cost- and clinical effectiveness of video-based SUD services.

The current work should be interpreted in the context of methodological limitations. First and foremost, the convenience sampling approach used by regional ATTCs was prone to both selection and response biases. Despite the distribution of the electronic survey to large regional mailing lists and a wide range of organizational settings, the final response rate was only 15%. Second, it is possible that organizations that were more comfortable with telehealth might also have been more comfortable completing an electronic survey of 100+ questions, which could have introduced systematic response bias. Third, our reliance on survey methodology limited our ability to gather in-depth feedback, given the commonly stated “survey fatigue” during the COVID-19 pandemic [39]. A more detailed and granular account of workforce perceptions could have been derived via a qualitative or mixed methods inquiry. Finally, it should be restated that trends in perceptions of telehealth services at SUD treatment and recovery organizations were examined from two separate cross-sectional survey samples. Thus, longitudinal changes in the opinions of specific individuals polled as representatives of the addiction workforce should not be inferred.

These limitations notwithstanding, the results of this study suggest the initially promising outlook for telehealth services among the addiction workforce, as reported by Molfenter et al [1] in 2021 shortly after the initiation of COVID-19 social distancing recommendations. This outlook is now followed by reasons for both optimism and concern following the discontinuance of those social distancing recommendations. Of potential concern, a smaller percentage of SUD organizations indicated current telehealth service availability following removal of COVID-19 social distancing recommendations. However, this might be expected as some organizations or professionals could only deliver remote services during the pandemic, whereas following discontinuation of safety distancing recommendations, organizations could provide services either in person or via telehealth. Encouragingly, the availability of telehealth services remains common and endorsed by representatives from most SUD organizations in this sample, as is generally consistent with the findings of a recent review [40]. Both video-based and telephonic modalities for telehealth services continue to be perceived positively, with health professionals finding video services more useful but telephone services easier. Taken together with other recent research concerning these specific telehealth modalities [41-44], it seems that salient setting-, patient-, and population-level matching considerations may be needed to promote useful—and equitable—access to telephone- and video-based telehealth services. Additionally, structural support is likely required to overcome other challenges in availing telehealth services related to cost, reimbursement, and patient privacy. These are significant issues that deserve greater attention in future research on telehealth services at SUD service organizations, as does determination of the relative cost- and clinical effectiveness of specific telehealth services.

## Abbreviations

ATTC: Addiction Technology Transfer Center
CFR: Code of Federal Regulations
MD: mean difference
REDCap: Research Electronic Data Capture
SAMHSA: Substance Abuse and Mental Health Services Administration
SUD: substance use disorder
TAM: Technology Acceptance Model
